# Preservation of Humoral Immunity and Response to Vaccination in Patients With IgAN Treated With Felzartamab

**DOI:** 10.1016/j.ekir.2026.106697

**Published:** 2026-07-09

**Authors:** Jonathan Barratt, Millie Shah, Nicholas S. Jones, Valeria Beckett, Jingxian Chen, Peishan Liu-Snyder, Uptal D. Patel, Donna L. Flesher, Brian M. Schwartz, Jürgen Floege

**Affiliations:** 1 Department of Cardiovascular Sciences, College of Medicine Biological Sciences and Psychology, University of Leicester, Leicester, UK; 2 Biogen, South San Francisco, California, USA; 3 Division of Nephrology and Cardiology, RWTH Aachen University, Aachen, Germany

**Keywords:** anti-CD38 antibody, anti-tetanus toxoid antibody response, anti-SARS-CoV-2 antibody response, COVID-19 vaccination, felzartamab (MOR202/TJ202/BIIB148), IgA nephropathy

## Introduction

IgA nephropathy (IgAN), the most common form of primary glomerulonephritis, is characterized by deposition of IgA-containing immune complexes in the kidney, resulting in glomerular injury and kidney dysfunction.^1^ Cluster of differentiation (CD)38^+^ plasma cells are the source of pathogenic antibodies (i.e., galactose-deficient IgA and anti-galactose-deficient-IgA1) hypothesized to drive disease.^2^ CD38^+^ plasma cells represent late, terminally differentiated stages of B-cell lineage that develop following antigen activation through both germinal center-dependent and -independent pathways.^3^^,^^4^ Unlike early B-lineage cells (e.g., naive or memory B cells), plasma cells secrete antibodies at a high rate, show reduced CD19 and CD20 expression, and have high CD38 expression.^3^

Targeting B-cell subsets directly (e.g., via CD19/CD20-directed depletion) or through maturation and survival cytokines, such as A proliferation-inducing ligand and B-cell activating factor, represents a promising treatment strategy across antibody-mediated diseases, including IgAN.^2^^,^^5^ These approaches affect early and late B-lineage populations and have been shown to impact humoral immunity, potentially increasing infection risk and decreasing vaccine response.^6^, ^7^, ^8^

Felzartamab is a human monoclonal antibody targeting CD38^+^ cells, including plasmablasts and plasma cells. In the phase 2 IGNAZ study, felzartamab reduced proteinuria and slowed estimated glomerular filtration rate decline versus placebo through month 24 (18 months posttreatment).^9^ Felzartamab treatment was associated with decreases in serum galactose-deficient-IgA1, IgA, IgG, and IgM during the 6-month treatment period. IgA reductions remained durable after end of treatment through month 24, whereas galactose-deficient-IgA1 increased toward baseline within ≈9 months and IgG and IgM recovered to baseline within 6 months of end of treatment.

In IGNAZ, infections observed with felzartamab treatment were grade 1 or 2 and not associated with the number of doses given.^9^ In preclinical studies using *ex vivo* human donor samples, felzartamab selectively depleted CD38^+^ antibody-secreting cells, including plasmablasts and plasma cells, while sparing earlier B-lineage cells.S1 Clinical biomarker assessment in IGNAZ confirmed that felzartamab treatment reduced circulating CD38^+^ plasmablasts without impacting earlier B-lineage cells.S2 These data suggest felzartamab may target key pathological drivers of IgAN while preserving important aspects of humoral immunity.

This exploratory analysis examined antigen-specific humoral responses in felzartamab-treated participants in IGNAZ (see Supplementary Methods). Protective IgG antibody titers against tetanus toxoid (anti-TT) and SARS-CoV-2 (anti-SARS-CoV-2) were evaluated at baseline and longitudinally after treatment. Antibody responses to reported COVID-19 infections were also assessed. A full protocol is provided in the Supplementary Methods.

## Results

**Figure 1:**
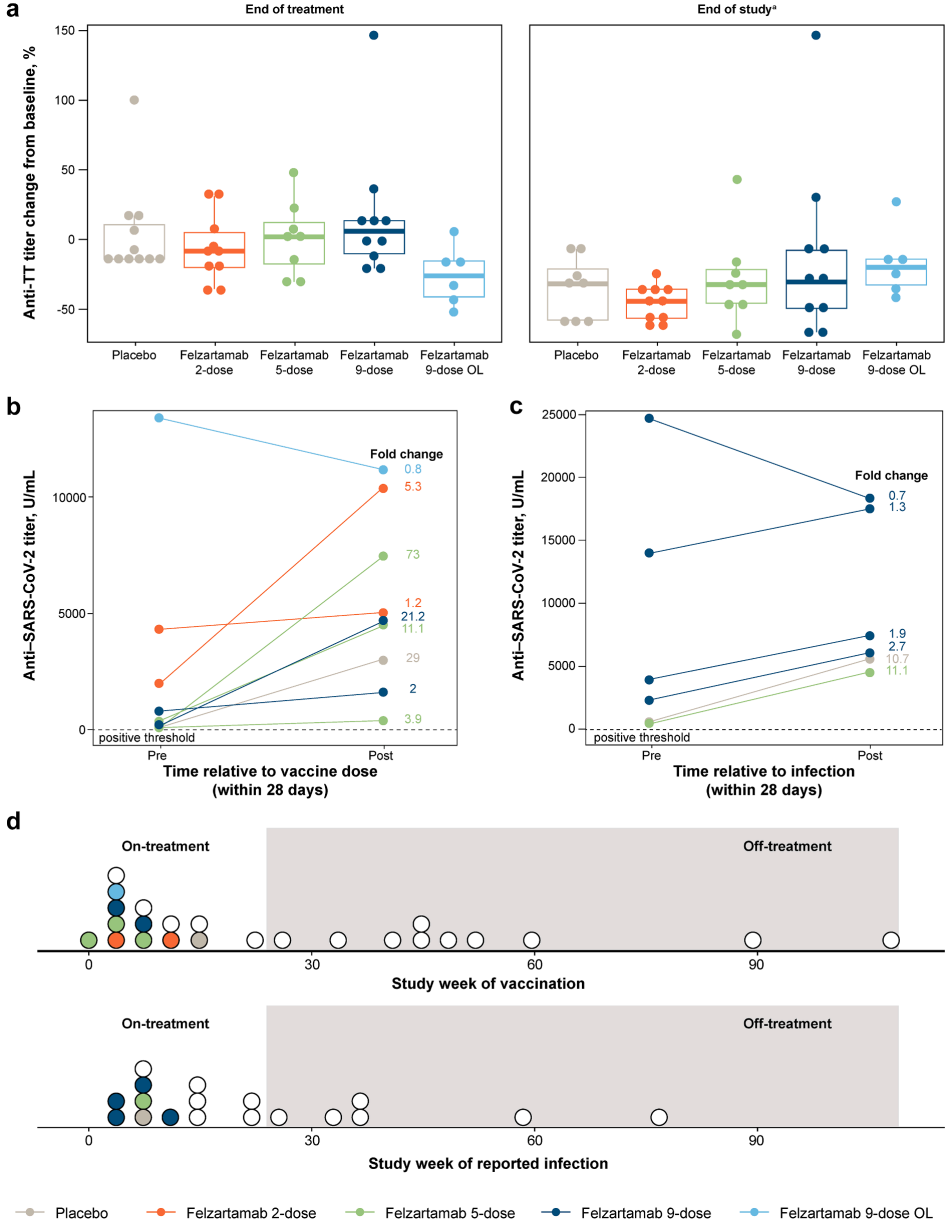
Changes in anti-TT and anti–SARS-CoV-2 antibody titers throughout the study, including (a) percentage change from baseline in anti-TT titers at end of treatment (6 months) and end of study (Part 1: 24 months, Part 2: 12 months), (b) change in pre- and post-vaccination anti–SARS-CoV-2 antibody titers for participants with available data, (c) change in pre- and post-infection anti–SARS-CoV-2 antibody titers, and (d) timing of SARS-CoV-2 vaccinations and infections. OL, open-label; SARS-CoV-2, severe acute respiratory syndrome coronavirus 2; TT, tetanus toxoid. ^a^End of study for Part 1 is at month 24 and for Part 2 is at month 12. In (b), 1 participant in the felzartamab 2-dose arm had two SARS-CoV-2 vaccination doses with pre-/post-vaccination titers available. Both are shown here as separate lines. In (c), all participants completed protocol-specified dosing. The total number of SARS-CoV-2 infections reported was 17, and 6 of 17 participants had pre- and postinfection data available (titers collected within 28 days of infection). In (d), circles represent vaccination (top) or infection (bottom) events. Colored circles correspond to events with pre- and post-event titer data available (represented in c), whereas open circles represent events without titer data available.

Of the 54 enrolled participants, 46 of 53 (87%) with available data had protective anti-TT titers at baseline (≥ 0.1 IU/ml; Table 1). Median baseline anti-TT titers ranged from 0.1 to 1.4 IU/ml across arms. Modest reductions in anti-TT titers were observed between end of treatment and end of study and were comparable between participants who received felzartamab and placebo (Figure 1a). Among those with protective baseline levels, 41 of 45 (91%) with longitudinal data maintained titers > 0.1 IU/ml on study. The lowest observed anti-TT titers across felzartamab treatment arms ranged from 0.08 to 0.69 IU/ml (Table 1). All 4 participants whose titers decreased to below protective levels had low baseline titers (< 0.2 IU/ml), and on-study reductions were minimal, with titers staying > 0.08 IU/ml. In 3 of these participants, anti-TT titers recovered by the last study observation with no reported booster vaccination. These data suggest felzartamab treatment was associated with modest and transient reductions in anti-TT titers, without clinically meaningful impairment of pre-existing long-lived immunity.

**Table 1:** Anti-TT immunity throughout the study

Anti-TT characteristics	Part 1				Part 2
	Placebo	Felzartamab 2-dose	Felzartamab 5-dose	Felzartamab 9-dose	Felzartamab 9-dose OL
Baseline anti-TT, IU/ml					
Participants at baseline, *n*	12	11	11	13	6
Mean (SD)	1.5 (1.3)	2.0 (2.4)	2.0 (4.1)	1.6 (1.3)	1.0 (2.0)
Median (IQR)	1.4 (0.4–1.9)	1.3 (0.21–2.6)	0.60 (0.24–1.4)	1.4 (0.6–2.7)	0.1 (0.07–0.57)
Lowest observed anti-TT, IU/ml					
Participants postbaseline, *n*	12	11	10	13	6
Mean (SD)	0.78 (0.60)	1.0 (1.4)	0.98 (1.4)	0.95 (0.90)	0.79 (1.7)
Median (IQR)	0.75 (0.23–1.2)	0.36 (0.11–0.98)	0.37 (0.19–0.72)	0.69 (0.46–1.4)	0.08 (0.04–0.28)
Participants with baseline anti-TT titers below protective levels (< 0.1), *n*	0	3	0	1	3
Participants with anti-TT titers decreased below protective levels (< 0.1) while on study, *n*^a^	1^b^	0	1	1^b^	1^b^

IQR, interquartile range; OL, open-label; TT, tetanus toxoid.

a All participants had low baseline titers (< 0.2 IU/ml). Reductions were minimal, and all participants maintained titers > 0.08 IU/ml.

b Participants’ titers recovered to protective levels by last observation without reported vaccination or booster.

The majority of participants received ≥ 1 COVID-19 vaccine within 12 months of baseline. Vaccine types included inactivated (*n* = 5), adenoviral (*n* = 6), and mRNA (*n* = 31); some participants received multiple types or doses. Among 48 participants with available data, 45 (94%) had positive baseline anti-SARS-CoV-2 titers (≥ 0.8 U/ml). Median baseline anti-SARS-CoV-2 titers ranged from 1464 to 15,681 U/ml across arms (Supplementary Table S1). Baseline positive anti-SARS-CoV-2 titers were maintained on study in all participants. The lowest observed postbaseline anti-SARS-CoV-2 titers ranged from 518 to 5939 U/ml across arms including placebo (Supplementary Table S2).

Sixteen participants received ≥ 1 SARS-CoV-2 vaccination on study (Supplementary Table S2), and 7 of 8 participants with pre- and postvaccine titers measured within 28 days of vaccination mounted a response regardless of treatment arm, suggesting preservation of vaccine-induced immunity (Figure 1b). The participant whose titers did not increase following vaccination had high antibody titers before vaccination, and postvaccination reductions were minimal (0.8-fold change). Seventeen participants experienced SARS-CoV-2 infections on study, with 1 experiencing 2 infections (Supplementary Table S2). Twelve infections occurred during the treatment period whereas 6 occurred during the off-treatment period (Figure 1d). Five of 6 participants with anti-SARS-CoV-2 titer data available pre- and postinfection generated antibody responses. The participant whose titers did not increase had high preinfection titers, and postinfection reductions were minimal (0.7-fold change). These observations occurred during the treatment period, suggesting that humoral antibody response to acute infection was preserved with felzartamab treatment (Figure 1c).

## Discussion

In this exploratory analysis of IGNAZ, pre-existing protective immunity to vaccines or infections was preserved with felzartamab treatment, as most participants maintained protective anti-TT and anti-SARS-CoV-2 immunity throughout the study. The mechanism of preservation of long-lived immunity is unclear but may relate to cellular characteristics, including CD38 expression, repopulation dynamics, and biophysical niche of antigen-specific cells.

Participants who received SARS-CoV-2 vaccination on study mounted antibody titers, indicating that vaccine-induced immunity was not compromised. Humoral immune responses to acute infection also remained intact, as 5 of the 6 participants who experienced infection on study developed antibody responses. In contrast, B cell–depleting agents that target earlier B-cell subsets, such as the anti-CD20 monoclonal antibody rituximab,S3 have been associated with impaired response to SARS-CoV-2 vaccination, resulting in increases in infection in patients with autoimmune diseases receiving anti-CD20 therapy.S4 Felzartamab differs in that it selectively targets mature CD38^+^ plasmablasts and plasma cells while preserving early B-lineage cells, enabling generation of new antibody-secreting cells in response to new vaccination or infection, which may explain the observed antibody responses to SARS-CoV-2 vaccination and infection. However, the relationship between measured anti-SARS-CoV-2 titers and protective immunity cannot be established from these data alone.

These analyses were exploratory and descriptive in nature and were limited by the small sample size and low number of evaluable vaccination and infection events, precluding formal statistical comparisons. Most evaluable vaccination and infection-associated antibody responses occurred on treatment and support preservation of humoral responses while receiving felzartamab. However, some infections occurred after treatment, but antibody titers were not available because of the timing of sampling, limiting interpretation of posttreatment responses that may reflect immune recovery while off treatment. Additionally, direct quantitative assessment of protective versus pathogenic CD38^+^ antibody-secreting cells is difficult, given their rarity in circulation; therefore, the study relied on antigen-specific antibody assessments. Neutralizing antibody activity or other functional measures of immunity were not assessed. Felzartamab is currently under investigation in IgAN in the phase 3 PREVAIL trial (NCT06935357), which will provide further insight into safety, including infection rates, in a larger population.

Felzartamab is also being evaluated in primary membranous nephropathy, which is thought to be driven by pathogenic anti-phospholipase A2 receptor autoantibodies produced by plasma cells.S5 In an analysis of the phase 1b/2a M-PLACE study in primary membranous nephropathy, felzartamab treatment was associated with robust immune responses to SARS-CoV-2 vaccination before or during treatment.S6 Antibody titers remained detectable throughout the study, and participants who developed COVID-19 recovered without medical intervention. Pathogenic anti- phospholipase A2 receptor autoantibodies were reduced to a greater extent than anti-TT antibodies, suggesting that protective immunity was selectively spared.S7 These findings are consistent with the current analysis from IGNAZ.

In conclusion, these findings suggest the potential for a favorable safety profile of felzartamab versus other B cell–targeting therapies that impact earlier B-cell populations. Results in IgAN align with previous observations in primary membranous nephropathy,S6^,^S7 suggesting a generalized mechanistic effect of maintaining protective humoral immunity when targeting CD38 with felzartamab in immune-mediated diseases.

## Disclosure

JB reports consulting and speaker fees from Alnylam, Argenx, Astellas, BioCryst, Calliditas, Chinook, Dimerix, Galapagos, Novartis, Omeros, Travere Therapeutics, Vera Therapeutics, Visterra, and Biogen; grant support from Argenx, Calliditas, Chinook, Galapagos, GSK, Novartis, Omeros, Travere Therapeutics, and Visterra; clinical trials with Chinook (ADU-CL-19 and ALIGN), Novartis (APPLAUSE), Omeros (ARTEMIS-IGAN), Visterra (ENVISION), Calliditas (NefIgARD), Vera Therapeutics (ORIGIN), and Biogen (IGNAZ); and research projects with Argenx, Calliditas, Chinook, Galapagos, GSK, Novartis, Omeros, Travere Therapeutics, and Visterra. MS, NSJ, VB, JC, PL-S, UDP, DLF, and BMS are employees of and may own stock in Biogen. JF has received lecture and/or consultancy honoraria from Alexion, AstraZeneca, Bayer, Biogen, Boehringer Ingelheim, Calliditas, CSL Vifor, Novartis, Otsuka, Roche, Stadapharm, Travere, Vera Therapeutics, and Vertex; has received support for attending meetings and/or travel from Stadapharm; and has participated on data safety monitoring boards for Novo Nordisk and Visterra.

## Supplementary Material

Supplementary file (PDF) **Supplementary Methods. Supplementary References. Table S1.** COVID-19 baseline vaccination rates and anti–SARS-CoV-2 titers. **Table S2.** Anti–SARS-CoV-2 immunity throughout the study.

## Footnotes

Supplementary file (PDF)

**Supplementary Methods.**

**Supplementary References.**
**Table S1.** COVID-19 baseline vaccination rates and anti–SARS-CoV-2 titers. **Table S2.** Anti–SARS-CoV-2 immunity throughout the study.
