# Supplementary material for: Preservation of Humoral Immunity and Response to Vaccination in Patients With IgAN Treated With Felzartamab
Source: Kidney Int Rep. 2026 Jul 9;11(10):106697. doi: 10.1016/j.ekir.2026.106697 (PMC13475335; doi:10.1016/j.ekir.2026.106697)
Supplement: Supplementary file 1 [file mmc1.pdf]

Supplementary Material

Supplementary Methods

The IGNAZ study (NCT05065970) was a 2-part, randomized, double blind, placebo-controlled phase 2a study to evaluate the efficacy and safety of felzartamab in 54 participants with biopsy-confirmed IgAN. Briefly, in Part 1, 48 participants were randomized 1:1:1:1 to placebo or IV felzartamab at 3 different dose regimens: 2 doses in 15 days, 5 doses in 2 months, or 9 doses in 5 months. In Part 2, 6 Japanese participants received the 9-dose regimen open-label. The primary endpoint was change in proteinuria as assessed by urine-protein creatinine ratio (UPCR) at 9 months, and secondary endpoints included change in eGFR and safety. Results from this study and detailed methods have been published previously.[9]

Serum samples were collected on study days 1 (baseline), 8, 15, 29, 57, 85, and 169 (end of treatment) during the on-treatment period and on days 267, 366, 548, and 730 during the off-treatment period. All evaluable samples were included in this analysis except for samples taken after initiation of prohibited concomitant medications. Antibody titers were analyzed using the Elecsys® Anti-SARS-CoV-2 S Assay (Roche Diagnostics, Indianapolis, IN) and VaccZyme™ Anti-Tetanus Toxoid IgG Enzyme Immunoassay MK010 (Thermo Fisher Scientific, Waltham, MA). Data were analyzed using R statistical analysis software version 4.2.1.

Supplementary Table S1. COVID-19 baseline vaccination rates and anti–SARS-CoV-2 titers

Supplementary Table S2. Anti–SARS-CoV-2 immunity throughout the study

Supplementary References

S1. Delfino TA, Ilieva KM, Landgraf K, et al. Felzartamab selectively and potently targets CD38+ antibody secreting cells from patients with immune-mediated kidney diseases. Poster presented at: American Society of Nephrology Kidney Week 2024; October 23-27, 2024; San Diego, CA.

S2. Shah M, Kivman L, Kräft T, et al. Felzartamab durably reduces disease relevant biomarkers through targeting of $CD38^+$ plasma cells and plasmablasts, the upstream drivers of IgA nephropathy (IgAN). Oral presentation at: American Society of Nephrology Kidney Week 2024; October 23-27, 2024; San Diego, CA. Abstract FR-OR69.

S3. Lafayette RA, Canetta PA, Rovin BH, et al. A randomized, controlled trial of rituximab in IgA nephropathy with proteinuria and renal dysfunction. *J Am Soc Nephrol* 2017;28(4):1306-13.

S4. Bitoun S, Henry J, Desjardins D, et al. Rituximab impairs B cell response but not T cell response to COVID-19 vaccine in autoimmune diseases. *Arthritis Rheumatol* 2022;74(6):927-33.

S5. Rovin BH, Ronco PM, Wetzels JFM, et al. Phase 1b/2a study assessing the safety and efficacy of felzartamab in anti-phospholipase A2 receptor autoantibody-positive primary membranous nephropathy. *Kidney Int Rep* 2024;9(9):2635-47.

S6. Rovin BH, Boxhammer R, Thakur A, et al. Immunologic responses after COVID-19 vaccination in patients with membranous nephropathy receiving anti-CD38 felzartamab therapy: Results from the Phase 1b/2a M-PLACE study. *Kidney Int Rep* 2022;7(9):2086-90.

S7. Flesher DL, Shah M, Boxhammer R, et al. Felzartamab reduces anti-PLA2R autoantibodies by selectively depleting $CD38^+$ plasma cells and plasmablasts, the main pathogenic cellular drivers of disease in primary membranous nephropathy (PMN). Poster presented at: American Society of Nephrology Kidney Week 2023; November 1-5, 2023; Philadelphia, PA.

Supplementary Table S1. COVID-19 baseline vaccination rates and anti–SARS-CoV-2 titers

	Part 1				Part 2	Total
	**Placebo (n=12)**	**Felzartamab 2-dose (n=12)**	**Felzartamab 5-dose (n=11)**	**Felzartamab 9-dose (n=13)**	**Felzartamab 9-dose OL (n=6)**	**(N=54)**
**Prior vaccination (within 12 months of baseline) by vaccine type, n[a]**						
Inactivated	2	1	0	2	0	5
Adenoviral	2	2	1	1	0	6
mRNA	5	7	8	7	4	31
Any	8	8	8	9	4	37
**Baseline levels of anti–SARS-CoV-2**						
Participants, n[b]	9	9	11	13	6	48
Participants negative (<0.8 U/mL) for anti–SARS-CoV-2 at baseline, n	0	1	0	1	1	3
Median (IQR), U/mL[c]	2926 (526-17,208)	3468 (2777-4863)	1464 (139-19,705)	4181 (1174-24,857)	15,681 (6727-32,232)	3701 (507-18,789)

BLQ, below the limit of quantification; IQR, interquartile range; mRNA, messenger RNA; OL, open-label; SARS-CoV-2, severe acute respiratory syndrome coronavirus 2; ULQ, upper limit of quantification.

[a] Some participants received multiple types of vaccines within 12 months of baseline.

[b] All participants with baseline data available. Baseline levels of anti–SARS-CoV-2 titers are missing for 6 participants.

[c] ULQ values imputed as 1.5×ULQ, and BLQ imputed as BLQ/1.5. Median values include participants considered positive and negative for anti–SARS-CoV-2 at baseline.

Supplementary Table S2. Anti–SARS-CoV-2 immunity throughout the study

	**Part 1**				**Part 2**
	**Placebo (n=12)**	**Felzartamab 2-dose (n=12)**	**Felzartamab 5-dose (n=11)**	**Felzartamab 9-dose (n=13)**	**Felzartamab 9-dose OL (n=6)**
**Lowest observed levels of anti–SARS-CoV-2 postbaseline**					
Participants, n[a]	9	9	10	13	6
Median[b] (IQR), U/mL	518 (206-956)	1019 (179-1911)	751.5 (89-4422)	1408 (221-3914)	5939 (1747-10,599)
**Participants who become negative (<0.8 U/mL) for anti–SARS-CoV-2 on study, n**					
	0	0	0	0	0
**Participants who experienced COVID-19 infection on study, n**					
	2	4	3	6	2
**Participants who received vaccination on study, n**					
	4	5	2	3	2

BLQ, below the limit of quantification; IQR, interquartile range; OL, open-label; SARS-CoV-2, severe acute respiratory syndrome coronavirus 2; ULQ, upper limit of quantification.

[a] All participants with data available (reported baseline and ≥1 postbaseline measurement), including those who experienced infections or received vaccines.

[b] ULQ values imputed as 1.5×ULQ, and BLQ imputed as BLQ/1.5.
